# In vivo confocal microscopy qualitative investigation of the relationships between lattice corneal dystrophy deposition and corneal nerves

**DOI:** 10.1186/s12886-021-02149-1

**Published:** 2021-12-27

**Authors:** Fengjiao Zhu, Ming Li, Chun Zhang, Chan Chen, Fangwei Ying, Danyao Nie

**Affiliations:** 1 Pudong New Area Eye and Dental Diseases Prevention & Treatment Center, Shanghai, 201399 P. R. China; 2 Department of Corneal and External Eye Diseases, Shenzhen Eye Hospital, Joint College of Optometry of Shenzhen University (Shenzhen University Health Science Center), Affiliated Shenzhen Eye Hospital of Jinan University, 18#, Zetian Road, Futian District, Shenzhen, 518040 P. R. China

**Keywords:** Lattice corneal dystrophy (LCD), In vivo confocal microscopy (IVCM), Corneal neurotropic phenomenon

## Abstract

**Background:**

To investigate the corneal neurotropic phenomenon in patients with lattice corneal dystrophy (LCD) with in vivo laser scanning confocal microscopy (IVCM).

**Methods:**

IVCM was performed on a total of 15 patients (28 eyes) with LCD annually at a follow-up. A collection of the data was acquired to be analyzed.

**Results:**

As indicated by the analysis, the LCD patients’ normal corneal stromal nerves (Grade 0) presented a decline with the prolongation of the follow-ups, corresponding to a gradual increase in grade I and II involving amyloid-wrapped nerve fibers, which demonstrated that the growing amount of amyloid deposit due to the corneal nerve invasion increased slowly over time.

**Conclusions:**

The neurotropic phenomenon could increase with its severity in the corneal lesion of the patients with LCD, and also reflect the distribution of the corneal nerves, to some extent. IVCM provides a rapid, noninvasive way to observe the corneal nerves, which can be an efficient means of better understanding the development of LCD.

**Supplementary Information:**

The online version contains supplementary material available at 10.1186/s12886-021-02149-1.

## Background

Corneal dystrophy (CD) is a type of bilateral non-inflammatory hereditary disease affecting the corneal central visual axis that becomes progressively opaque. The disease progresses slowly, having little or no relationship with the systemic or environmental factors. CD has characteristic pathological changes [1, 2]. Lattice corneal dystrophy (LCD) is a hereditary disease in which the amyloid deposits in the cornea, causing lattice-like opacities in the cornea with a detrimental effect on visual acuity [3]. LCD affects males and females equally, with age of onset varying as described in history and physical [4]. LCD is known to have five subtypes: LCDI, II, III, IIIA, IV; in LCDI, IIIA, and IV, mutations in the BIGH3 gene can result in amyloid deposition in the corneal stroma, but no such pathological changes are observed in other tissues [5].

LCD Type I and IC3D described variants such as Granular Corneal Dystrophy Type I (GCD1), Granular Corneal Dystrophy Type II (GCD2), a.k.a. Avellino Corneal Dystrophy, Thiel-Behnke Corneal Dystrophy (TBCD), and Reis-Bückler Corneal Dystrophy (RBCD), caused by the mutation of human transforming growth factor β-induced (TGFB-I) gene located on chromosome 5 (genetic locus 5q31). These are categorized as the “superfamily” which are all autosomal dominant. TGFB-I induced protein (TGFB-I p) is a 68 kDa protein also called “keratoepithelin” or “Big-h3” that is found in the extracellular matrix of several human tissues, particularly abundant in the cornea. Mutations of TGFB-I gene encoding for TGFB-I p are associated with variable protein aggregation and deposition (amyloid and nonamyloid aggregates) in the cornea [1].

In vivo corneal laser scanning confocal microscopy (IVCM) is helpful to evaluating the morphological characteristics of corneal dystrophies at the histological level and maybe helpful in diagnosis and understanding the pathophysiology of disease [6]. It is well known that the phenotypic spectrum, also at IVCM beyond slit lamp, of genetically confirmed granular and LCD corneal dystrophies in patients may change over time [7]. Mazzotta C et al. just reported in the literature that corneal deposits started at sub-Bowman level, often surrounding the subepithelial plexus nerve fibres, being present in the deeper stromal layers until 250–300 μm of depth” ref. above mentioned.

We observed the corneal nerves were wrapped by amyloid deposits in a gradual fashion, and the entire involvement of the corneal neural network occurred eventually; thus this condition was defined as a neurotropic phenomenon. Since a direct neural invasion by amiloid was never demonstrated, indeed the stroma is invaded by deposition, but a secondary neural structures disorganization and alteration by amylod deposition was documented surrounding and dislocating the nerves fibres reflecting their distribution, which indicated the time-dependent growing amount of amyloid deposit due to the corneal nerve invasion increased slowly over time. The aim of this study was to observe the neurotropic phenomenon in the corneas of the patients with LCD using IVCM, which could provide us with a new thinking of treatment to prevent the corneal lesions.

## Methods

### Patients

The study was conducted in compliance with informed consent regulations and the Declaration of Helsinki; the protocol was approved by the Internal Review Board (IRB) of Shenzhen Eye Hospital; and informed consents were obtained from the patients with LCD, who numbered 15 with eligible 28 eyes for the study in Shenzhen Eye Hospital in Shenzhen of China during the period from March 2009 to March 2018. The group of subjects was composed of 7 women and 8 men aged 35.9 ± 4.36 y, including 2 patients monocular.

**Fig. 1:**
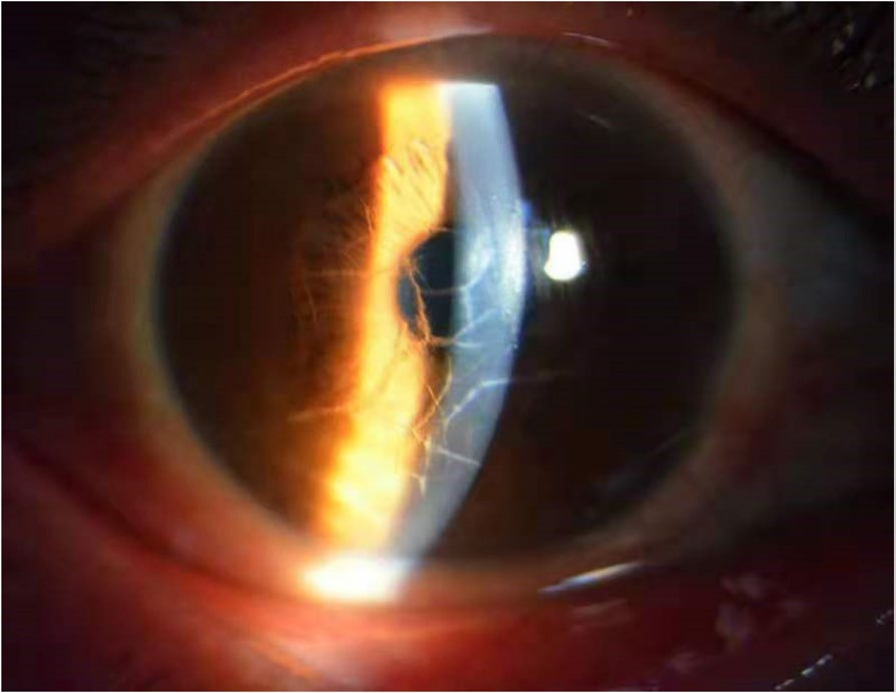
Photograph of the anterior segment of LCD, the corneal stroma seen in lattice-like stripe turbidity

All patients underwent the conventional slit lamp examination by an ophthalmologist, the corneal stroma seen as lattice-like streaky turbidity, as indicated by the photograph of the anterior segment of the patient’s eye with LCD (Fig. 1). A differential diagnosis was made to exclude other types of corneal dystrophy, glaucoma, uveitis, keratitis, corneal ulcer, conjunctivitis and leukoplakia. After the first examination, all the patients would undergo IVCM annually at a follow-up. Respectively, 8 patients (16 eyes) received the examination for 10 consecutive years; 5 patients (9 eyes), for 6 consecutive years; and 2 patients (3 eyes), for 4 consecutive years. The data collected covered the medical and ophthalmological history including age, age at diagnosis, gender, detailed slitlamp examination and IVCM imaging. Without exception, every individual was informed of the aim of each data recording.

### IVCM

The area in contact with the cornea was examined using IVCM with magnification up to X800 (HRT II Rostock Cornea Module, diode laser 670 nm, Heidelberg Engineering GmbH, Germany), the images consisting of 384 × 384 pixels covering an area of 400 × 400 mm with a transverse optical resolution of approximately 1 mm/pixel and an acquisition time of 0.024 s (Heidelberg Engineering, Germany). After that, the patient’s cornea was routinely examined. To standardize the measurements, all images were subsequently randomized and encoded by a single independent observer.

The position where we take the point is within the range of 2 mm in the central diameter of each quadrant and every 100um vertical depth. Then the stromal nerves were captured and processed by IVCM.

### Image analysis

**Fig. 2:**
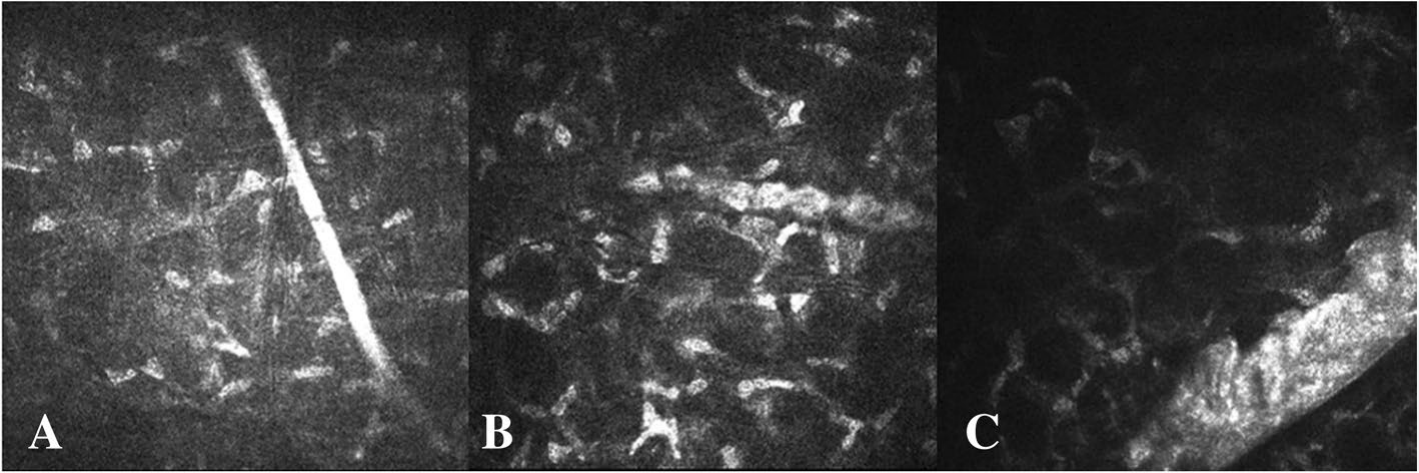
**A** The first type of LCD that the corneal stromal nerves not involved, the nerve fibers straight, and highly reflective, with smooth borders and a straight structure. **B** Some of amyloid encapsulating the nerve fibers of the corneal stroma; the affected nerve fibers becoming thickened and the unwrapped nerves growing thinner, taking the form of being beaded, defined as Grade I of neurotropic phenomenon. **C** A large number of amyloids in the nerve fibers enveloping the corneal stroma; the affected nerve fibers significantly thickened, defined as Grade II of neurotropic phenomenon of LCD

**Fig. 3:**
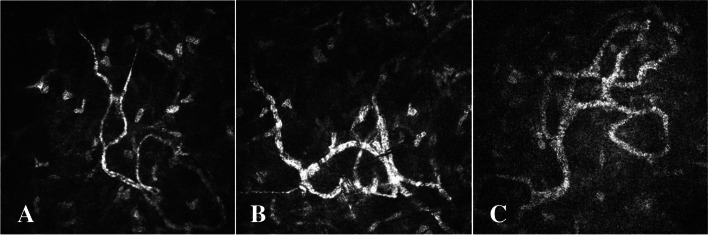
**A** The second type of LCD that the corneal stroma nerves not involved; the curved nerve fibers having a fine, highly reflective, slender, curved structure. **B** The curved nerve fibers partially thickened due to LCD lesions, showing uneven thicknesses; the curved nerve fibers not affected in the lower left, still defined as Grade I of neurotropic phenomenon of LCD. **C** The nerve fibers of the corneal stroma significantly thickened after being wrapped by LCD lesions, defined as Grade II of neurotropic phenomenon of LCD

**Fig. 4:**
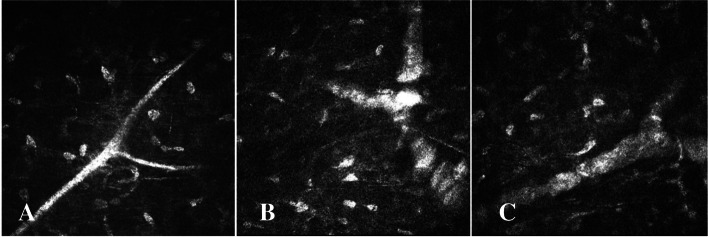
**A** The third type of LCD that the corneal stroma not involved, showing branching nerve fibers in the corneal stroma. **B** Some of amyloid encapsulating the branching nerve fibers in the corneal stroma, with the nerve fibers becoming thickened and segmented, defined as Grade I of neurotropic phenomenon of LCD. **C** A large number of amyloids wrapping the nerve fibers in the corneal stroma, with the nerve fibers becoming all significantly thickened, without segmentation, defined as Grade II of neurotropic phenomenon of LCD

For the convenience of description, the corneal stromal nerve fibers that were not affected by LCD lesions were divided into three categories according to the pathway and thickness of the nerve in the corneal stroma by IVCM: The first type was composed of the straight nerve fibers (Fig. 2A); the second type, of the curved, thinner nerve fibers (Fig. 3A); and the third type, of the branching, thicker nerve fibers (Fig. 4A).

#### Neurotropic grades

##### Grade 0

As indicated by the IVCM, the corneal stromal nerve fibers, which were not affected by the lesion, had a highly reflective strip-like structure running continuously, with the boundary being smooth and the structure being clear. This was defined as Grade 0 of the neurotropic phenomenon (Figs. 2A, 3A and 4A). As shown in Fig. 2A, the corneal stroma nerves were not involved in LCD lesions, showing a clear structure, smooth border and highly reflective strip-like structure running straight. As shown in Fig. 3A, the corneal stroma nerves of LCD were not involved, relatively curved and slender, the structure being highly reflective and its boundary being clear. Moreover, the corneal stroma nerves of LCD were not involved, showing an obvious branching structure, whereas the nerve fibers were relatively coarse (Fig. 4A).

##### Grade I

Part of the agglomerate and high-reflective structure wrapped the corneal nerve, the corneal nerve slightly thickened, beaded or segmental, which was defined as Grade I of the neurotropic phenomenon (Figs. 2B, 3B and 4B). Some of the amyloid-coated nerve fibers of the corneal stroma were thickened by the affected nerve fibers, while the unwrapped nerves were thinner, thus resulting in beaded nerve fibers (Fig. 2B). The bent and curved nerve fibers were partially thickened by the LCD lesions, presenting uneven thicknesses (Fig. 3B). There were curved nerve fibers that are not affected in the lower left. Some of the amyloid-coated nerve fibers were bifurcated in the corneal stroma, the nerve fibers being segmental (Fig. 4B).

##### Grade II

A large number of agglomerate and highly reflective structures wrapped the corneal nerve, with the corneal nerve significantly thickened. This neurotropic phenomenon of LCD was defined as Grade II (Figs. 2C, 3C and 4C). In the nerve fibers a large number of amyloids enveloped the corneal stroma, the affected nerve fibers significantly thickened, which was defined as Grade II of the neurotropic phenomenon of LCD (Fig. 2C). When wrapped by LCD lesions, the nerve fibers of the corneal stroma became significantly thickened, which was defined as Grade II of neurotropic phenomenon of LCD (Fig. 3C). Additionally, when a large number of amyloids wrapped the nerve fibers in the corneal stroma, the nerve fibers were all significantly thickened without segmentation, which was also defined as Grade II of the neurotropic phenomenon of LCD (Fig. 4C).

The grading of all the pictures was performed by two observers. The consistent results were used.

### Statistical methods

The data were analyzed using IBM SPSS Statistics for Windows, Version 19.0(IBM Corp, Armonk, NY, USA) and reported as mean ± SD.

## Results

**Fig. 5:**
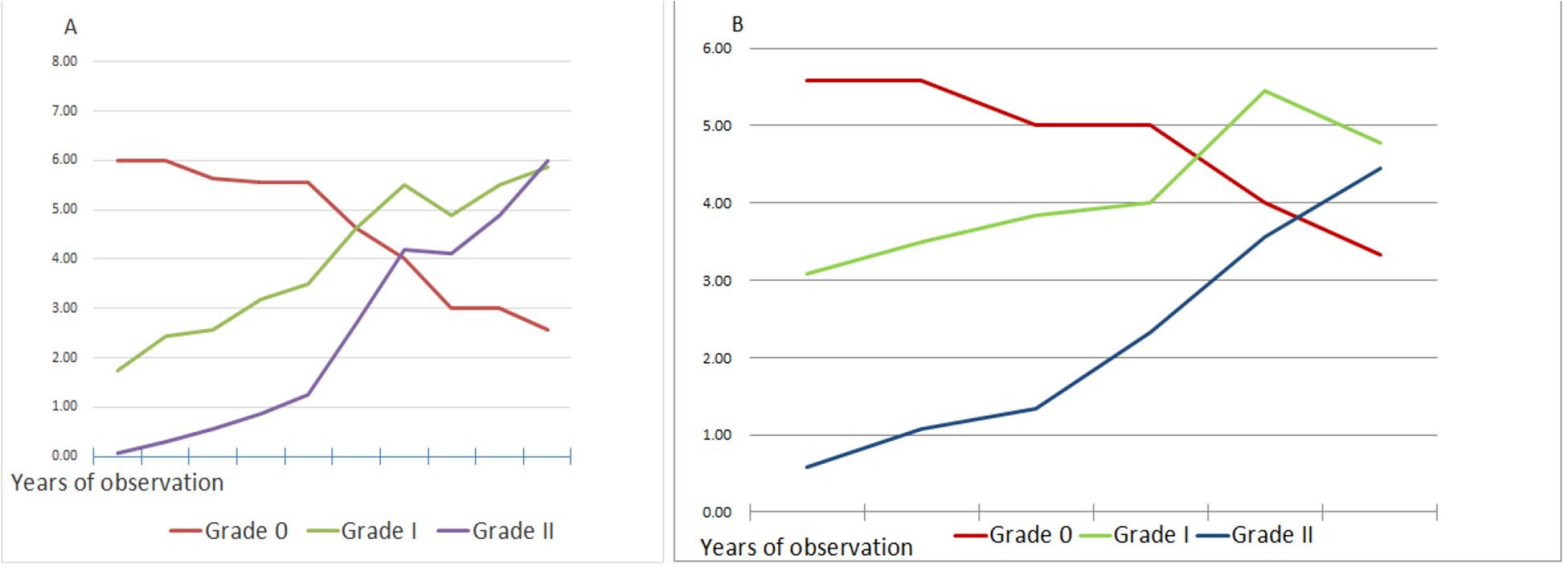
**A** The trend of 16 eyes of neurotropic phenomenon of LCD based on a 10-year observation. **B** The trend of the remaining patients of neurotropic phenomenon of LCD

At each follow-up examination with IVCM, the cornea was divided into 4 quadrants, each taken once every 100um depth. According to the criteria, the nerve fibers were graded, and each nerve structure found was counted 1 as follows:

As shown in Table 1, 8 patients (16 eyes) underwent a 10-year observation, Out of the corneal neuropathic data averaged came a trend chart, as shown in Fig. 5A. With the prolongation of the observation period, the nerves of Grade 0 as normal presented a gradual decrease, while Grade I and II nerves affected, the amyloid-encapsulated nerve fibers, did a gradual increase.

**Table 1:** The 16 eyes’ classification of the neurotropic phenomenon of LCD based on a 10-year observation

Eye No.	Part 1: Years of observation														
	1st year			2nd year			3rd year			4th year			5th year		
	Classification of neurotropic phenomenon of LCD														
	Grade 0	Grade I	Grade II	Grade 0	Grade I	Grade II	Grade 0	Grade I	Grade II	Grade 0	Grade I	Grade II	Grade 0	Grade I	Grade II
1	6	2	0	6	2	0	5	2	0	5	4	1	4	4	1
2	8	2	0	9	2	0	8	3	1	7	2	1	7	5	2
3	5	1	0	6	3	1	5	3	1	5	3	1	6	3	1
4	4	2	1	6	3	1	5	3	1	5	3	1	6	3	1
5	4	3	0	4	2	0	3	2	0	4	2	0	4	4	1
6	3	2	0	3	2	0	3	4	0	4	5	1	4	5	1
7	8	1	0	8	3	1	7	3	1	7	3	1	8	3	3
8	7	1	0	6	3	0	7	2	0	7	4	0	6	3	1
9	5	2	0	4	2	0	5	2	1	5	2	1	6	2	1
10	6	2	0	6	2	0	5	2	0	5	2	1	4	2	1
11	9	1	0	8	1	1	8	1	1	7	4	1	7	4	1
12	7	1	0	6	4	0	7	4	0	6	5	1	6	5	1
13	4	1	0	5	4	1	4	2	1	4	2	2	3	3	2
14	8	4	0	7	3	0	7	2	0	7	3	0	8	3	1
15	5	1	0	6	1	0	4	4	1	4	4	1	5	4	1
16	7	2	0	6	2	0	7	2	1	7	3	1	5	3	1
Average	6.00	1.75	0.06	6.00	2.44	0.31	5.63	2.56	0.56	5.56	3.19	0.88	5.56	3.50	1.25

Eye No.	Part 2: Years of observation														
	6th year			7th year			8th year			9th year			10th year		
	Classification of neurotropic phenomenon of LCD														
	Grade 0	Grade I	Grade II	Grade 0	Grade I	Grade II	Grade 0	Grade I	Grade II	Grade 0	Grade I	Grade II	Grade 0	Grade I	Grade II
1	4	4	2	3	7	3	3	6	5	2	7	6	2	9	6
2	6	4	3	5	8	5	4	7	6	5	8	7	4	8	9
3	4	3	2	5	5	3	4	4	3	3	5	4	2	5	6
4	4	3	2	5	5	3	4	4	3	3	5	4	2	5	6
5	2	4	1	3	4	2	2	4	4	1	4	4	1	4	4
6	3	5	4	4	6	3	3	3	3	2	6	3	2	5	3
7	7	3	3	5	4	5	3	7	6	4	4	7	4	4	8
8	5	6	4	4	5	5	3	6	5	4	5	6	3	7	6
9	4	5	4	3	5	4	3	4	4	2	5	4	1	5	4
10	5	7	1	3	6	5	2	3	5	4	6	3	4	6	6
11	6	5	5	6	5	3	3	5	3	3	5	5	2	5	8
12	5	6	2	4	4	6	3	6	3	2	4	6	2	5	6
13	4	3	2	2	4	5	3	5	5	2	4	5	3	6	5
14	6	7	3	5	5	5	4	6	2	5	5	6	5	5	7
15	3	4	2	3	8	6	2	5	6	3	8	5	1	8	6
16	6	5	3	4	7	4	2	3	3	3	7	3	3	7	6
Average	4.63	4.63	2.69	4.00	5.50	4.19	3.00	4.88	4.13	3.00	5.50	4.88	2.56	5.88	6.00

As indicated in Table 2, the data of the neurotropic phenomenon of LCD were acquired from the remaining patients, 9 undergoing 6-year follow-ups and 2 (3 eyes) from 2 having 4-year follow-ups. The trend chart generated as shown in Fig. 5B, although the observation period was shorter than that as indicated in Fig. 5A, the nerves of Grade 0 as normal presented a gradual decrease, while those of Grade I and II, the amyloid-encapsulated nerve fibers, did a gradual increase in the trend; However, the slope was smaller than that as indicated in Fig. 5A, considering the shorter period of observation.

**Table 2:** Data of the neurotropic phenomenonof LCD from the remaining 12 patients, 9 with a 6-year of observation and 2 (3 eyes) with a 4-year observation

Eye No.	Years of observation																	
	1st year			2nd year			3rd year			4th year			5th year			6th year		
	Classification of neurotropic phenomenon of LCD																	
	Grade 0	Grade I	Grade II	Grade 0	Grade I	Grade II	Grade 0	Grade I	Grade II	Grade 0	Grade I	Grade II	Grade 0	Grade I	Grade II	Grade 0	Grade I	Grade II
1	5	4	1	4	4	1	3	4	1	4	4	2	3	7	3	4	6	5
2	7	2	1	7	5	2	6	4	2	6	4	3	5	8	5	5	5	6
3	5	3	1	6	3	1	4	3	1	4	3	2	5	5	3	3	5	3
4	7	4	1	6	4	1	6	5	2	5	6	3	4	6	4	4	7	4
5	4	2	0	4	4	1	3	3	1	2	4	1	3	4	2	2	4	4
6	4	5	1	4	5	1	3	5	1	3	5	4	4	6	3	3	5	3
7	7	3	1	8	3	3	6	3	3	7	3	3	5	4	5	3	4	6
8	7	4	0	6	3	1	6	5	2	5	6	4	4	5	5	4	3	5
9	3	2	0	3	4	0	4	5	1	4	5	1	3	4	2	2	4	4
10	8	3	1	7	3	1	7	3	1	8	3	3						
11	6	3	0	7	2	0	7	4	0	6	3	1						
12	4	2	0	5	2	1	5	2	1	6	2	1						
Average	5.58	3.08	0.58	5.58	3.50	1.08	5.00	3.83	1.33	5.00	4.00	2.33	4.00	5.44	3.56	3.33	4.78	4.44

## Discussion

LCD, a clinically common hereditary disease, can lead to severe visual impairment and significant heritability. The condition is characterized by the deposition of amyloid in the cornea leading to the appearance of corneal stroma. These deposits create linear, lattice-like opacities arising primarily in the central cornea, while the peripheral cornea is often spared [8]. When illuminated by post-illumination, the lattice lines and nodules are visible double-profiles with an optically transparent core that can be directed to the periphery (Generally, it does not reach the limbus of the cornea) and deep expansion of the matrix; it can also stretch the epithelial layer to make the surface of the corneal epithelium irregular [9].

We performed IVCM on the cornea of LCD patients at each follow-up. As suggested by Fig. 2A-C, the amyloid deposit could have an increasing effect on the coarser nerve fibers running straight through the corneal stroma; the straight nerve fibers could not be affected by the lesions (Fig. 2A); as the condition progressed, some of amyloid-like nerve fibers could have a tendency to be wrapped, with the nerves becoming beaded (Fig. 2B); and at the late stage of the lesion, a large amount of amyloid could encapsulate the nerve fibers, with the nerves becoming significantly thickened (Fig. 2C).

As suggested by the series of Fig. 3A-C, the amyloid deposit, could affect the finer nerve fibers that were bent in the corneal stroma; the nerve fibers that were bent finely could not be affected by the lesions (Fig. 3A); as the condition progressed, a small amount of amyloid-like nerve fibers, with time, could be wrapped, with the nerves irregularly ringed (Fig. 3B); and at the late stage of the lesion, a large amount of amyloid could encapsulate the nerve fibers, with the nerves becoming significantly thicker and curved, and with multiple flower rings (Fig. 3C) which could be connected into a mass, if they continued to develop.

As suggested by the series of Fig. 4A-C, the amyloid deposit could have a tendency to affect the large corneal nerve branches; the lesion was unlikely to affect the branching of the corneal stroma, and the coarser nerve fibers (Fig. 4A); as the condition progressed, some of amyloid-like nerve fibers could grow to be wrapped, the nerves becoming segmental (Fig. 4B); and at the late stage of the lesion, a large amount of amyloid was likely to encapsulate the nerve fibers, the nerves becoming significantly thickened under the confocal microscope (Fig. 4C).

Although there has been a recent trend toward quantitative studies, using IVCM, readily reproducible methods and reference values for quantitative assessment still need to be standardized [10]. Most studies report lower interobserver repeatability compared with intraobserver repeatability, and observer experience is known to be an important factor [11]. We defined this phenomenon as neurotropic phenomenon that amyloid deposits could wrap along with the corneal nerve in LCD patients, eventually involving the entire corneal neural network. By using IVCM to observe the cornea of LCD patients at follow-ups for years, we could better understand the development of the lesion. While conventional light microscopes are limited by light scatter from structures outside of the focal plane, IVCM creates a point source of light by a pinhole aperture, focused by an objective lens on the tissue, which can well facilitate the observation of the structure of living lesions in LCD patients [12]. Since the slit lamp examination shows no pathologic changes or mild lattice-like corneal turbidity at the early stage, corneal lesions and neurotropic phenomenon with mild degree could be observed by using IVCM, which will be better for early diagnosis [13]. As previously reported, the progresses of corneal lesions in LCD patients could actually reflect the distribution of nerve fibers in the corneal stroma to some extent [14].

According to the trend charts (Fig. 5A & B), the normal corneal stromal nerve, defined as Grade 0, could have a tendency to deteriorate during the long-term observation. Moreover, the affected nerve wrapped in amyloid, defined as Grade I and II, could present a gradual increase. The statistical results also suggested that the corneal nerve invagination due to amyloid deposits in LCD patients tends to aggravate over time. In view of the findings, it can be hypothesized that the mechanism of corneal lesion in LCD refers to the nerve fibers in the corneal stroma which are wrapped and thickened, with their density increased over time.

As reported by Mazzotta C et al. [7], the IVCM analysis provided an interesting insight in the microstructure of the deposits showing fusiform or linear deposits of amyloid origin, starting at sub-Bowman layers and involving the anterior-mid stroma until 300–350 μm of depth. The amyloid component is typically only apparent by histopathology and does not resemble lattice lines. However, despite the accurate morphological and clinical analysis, the genetic diagnostic testing represents the most important tool for certain diagnosis both in common and atypical evolving phenotypic spectra. The natural history of these disorders is progression of the corneal deposition throughout life. Progression is faster in homozygous cases. The phenotypic spectrum of genetically confirmed LCD in patients may change over time with a transforming clinical appearance and evolving in the adulthood with different prognosis and therapeutic responses among these variants.

As shown in Fig. 5A, the observation time of 4.5 year is the break point whereas the time is 3.5 year in Fig. 5B. The Grade 0 did a faster decrease and Grade I and II showed a faster increase after the break point time. This trend remind us to inform patients that the cornea lesions will aggravates after 3 ~ 4 years, which need a shorter regular visits to hospital, and corneal transplantation will be needed in severe cases.

Limitations of this study included the small sample size and inconsistency of observation time, which resulted in wide 95% CIs that could be reduced with a larger number of patients. Therefore, more LCD patients are needed to observe for a longer period of time. The extraction and identification of neurotropic substances can be of great benefit to the design of anti-neuronal drugs for clinical treatment of LCD patients, which may prevent the corneal lesions.

## Conclusions

IVCM provides a rapid, minimally invasive (eye contact examination) way to observe the corneal nerves, which can be an efficient means of better understanding the neurotropism of LCD lesions. The neurotropic phenomenon could increase with its severity in the corneal lesion of the patients with LCD, and also reflect the distribution of the corneal nerves, to some extent. Whether the neurotropic phenomenon of LCDs with different gene phenotypes are identical, etc., further research is merited. Ideally, the current study can help us conduct in-depth researches in this direction.

## Supplementary Information

**Additional file 1.**

## Data Availability

The datasets used and/or analyzed during the current study are available from the corresponding author on reasonable request.

## Abbreviations

LCD: Lattice corneal dystrophy
IVCM: In vivo confocal microscopy
GCD1: Granular Corneal Dystrophy Type I
GCD2: Granular Corneal Dystrophy Type II
TBCD: Thiel-Behnke Corneal Dystrophy
RBCD: Reis-Bückler Corneal Dystrophy
TGFB-I: Transforming growth factor β-induced
TGFB-I p: TGFB-I induced protein
IRB: Internal Review Board
